# Supplementary material for: Building Sub-Saharan African PBPK Populations Reveals Critical Data Gaps: A Case Study on Aflatoxin B1
Source: Toxins (Basel). 2025 Oct 3;17(10):493. doi: 10.3390/toxins17100493 (PMC12567995; doi:10.3390/toxins17100493)
Supplement: Supplementary file 1 [file toxins-17-00493-s001.zip › toxins-3841193-supplementary.pdf]

Supplementary Materials: Building Sub-Saharan African PBPK Populations Reveals Critical Data Gaps: A Case Study on Aflatoxin B1

Orphélie Lootens, Marthe De Boevre, Sarah De Saeger, Jan Van Bocxlaer and An Vermeulen

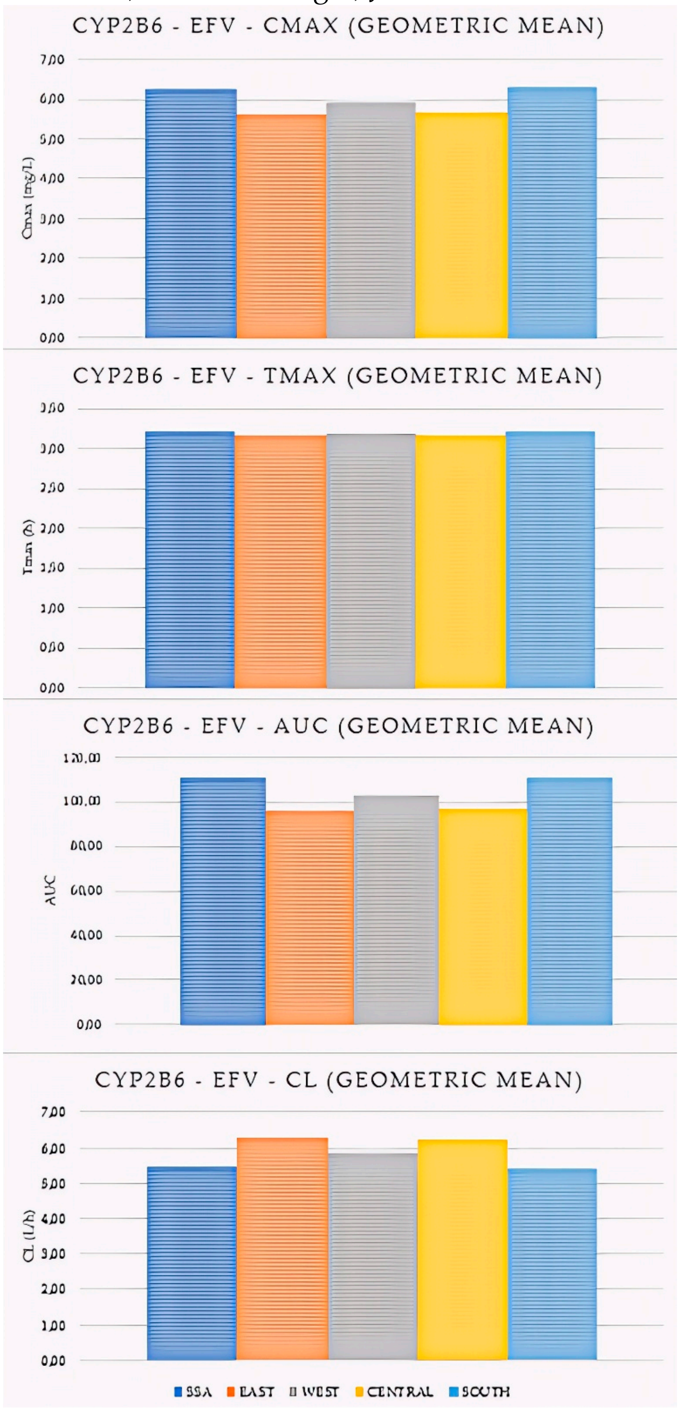

Figure S1: Representation of the pharmacokinetic (PK) parameters C_{max}, T_{max}, AUC_{ss} and CL/F of efavirenz (EFV) after a standard dose of 600 mg EFV o.d. for 30 days. The PK parameters are shown in bar charts representing the 5 regions being Sub Saharan Africa (SSA), East, West, Central and South Africa.

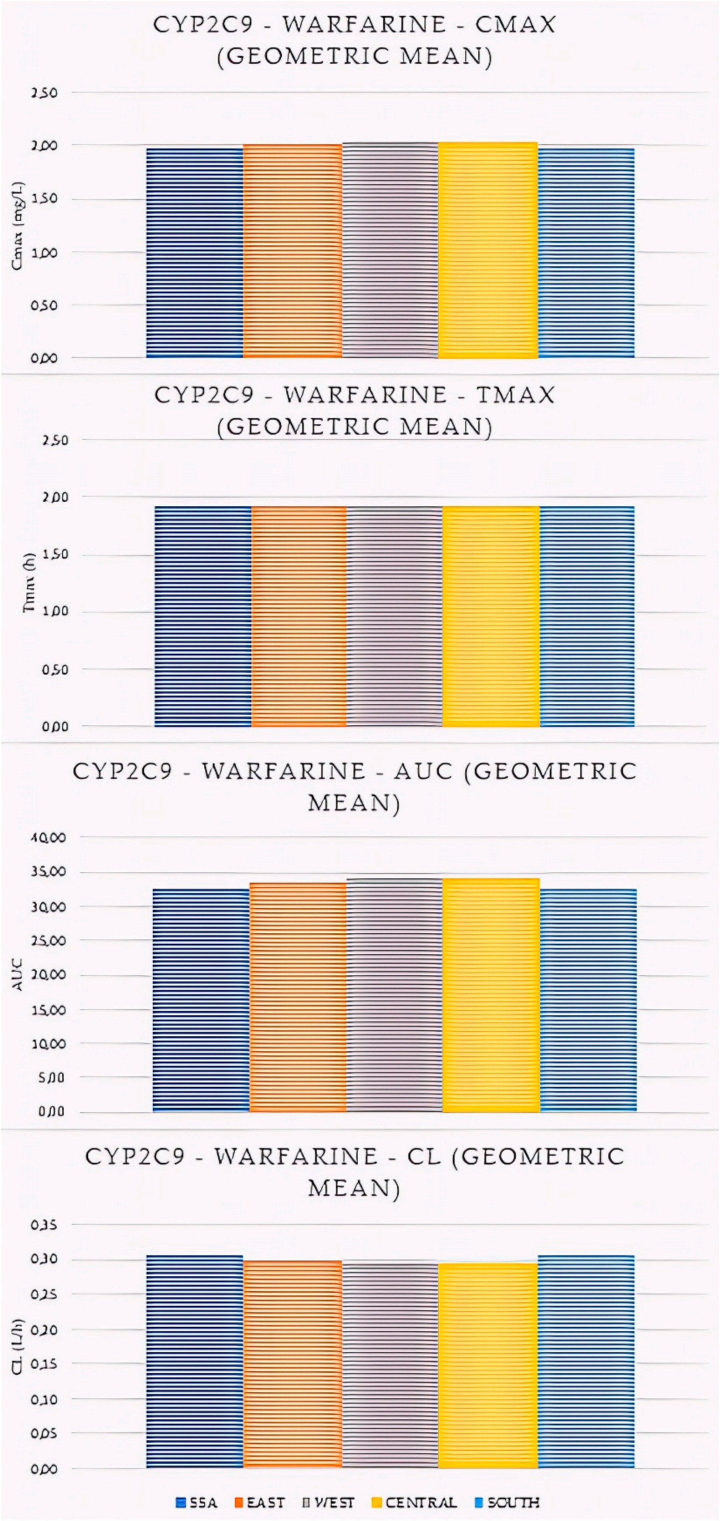

Figure S2: Representation of the pharmacokinetic (PK) parameters C_{max}, T_{max}, AUC_{ss} and CL/F of warfarine after a standard dose of 10 mg o.d. for 30 days. The PK parameters are shown in bar charts representing the 5 regions being Sub Saharan Africa (SSA), East, West, Central and South Africa.

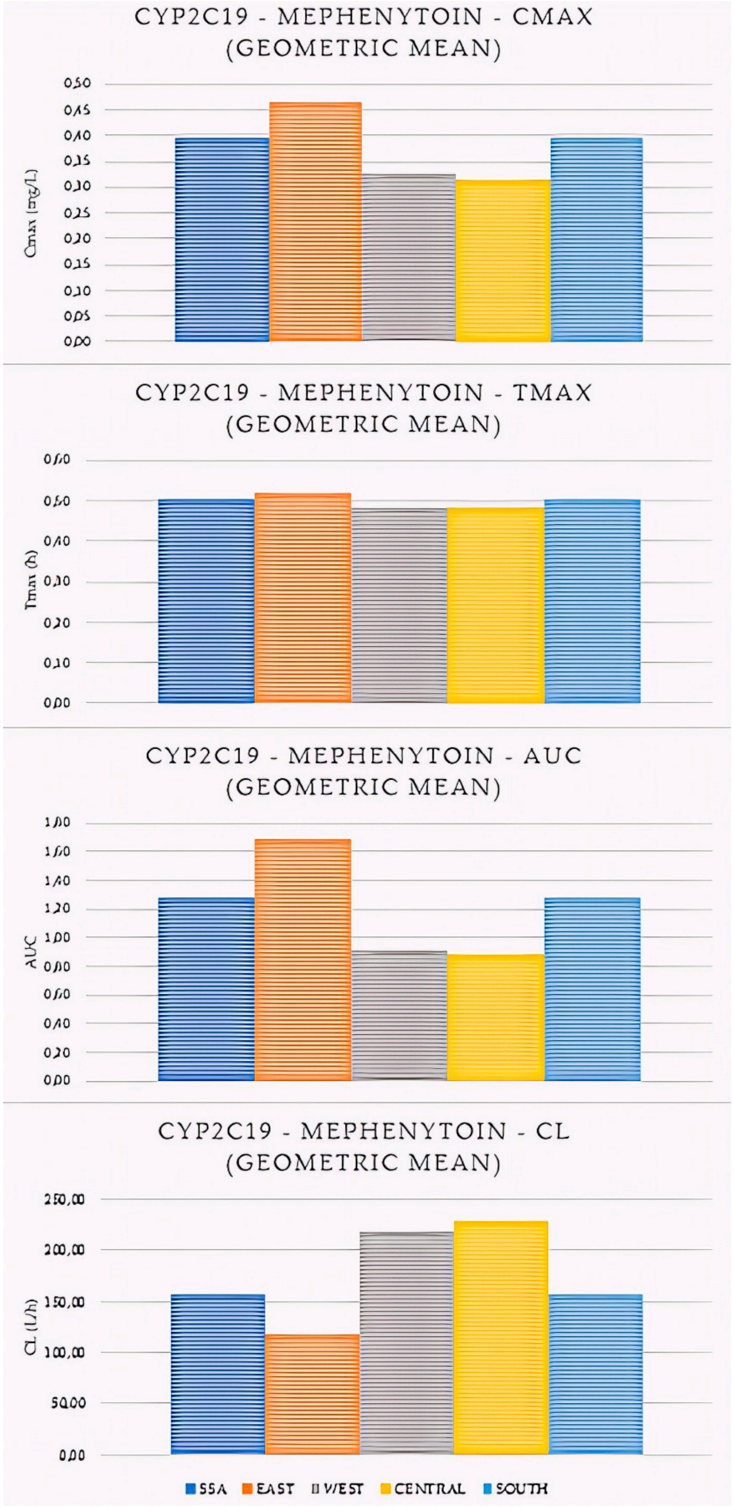

Figure S3: Representation of the pharmacokinetic (PK) parameters C_{max}, T_{max}, AUC_{ss} and CL/F of mephenytoin after a standard dose of 200 mg o.d. for 30 days. The PK parameters are shown in bar charts representing the 5 regions being Sub Saharan Africa (SSA), East, West, Central and South Africa.

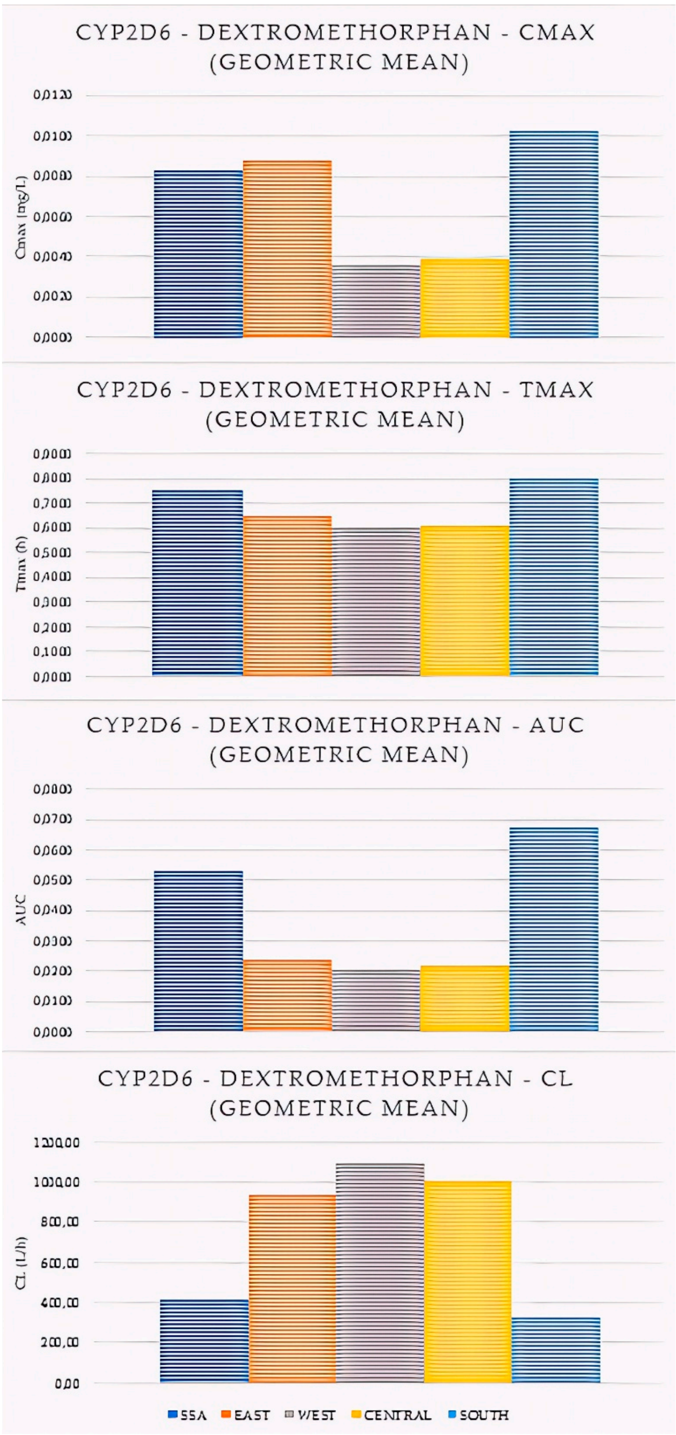

Figure S4: Representation of the pharmacokinetic (PK) parameters C_{max}, T_{max}, AUC_{ss} and CL/F of dextromethorphan after a standard dose of 22 mg o.d. for 30 days. The PK parameters are shown in bar charts representing the 5 regions being Sub Saharan Africa (SSA), East, West, Central and South Africa.

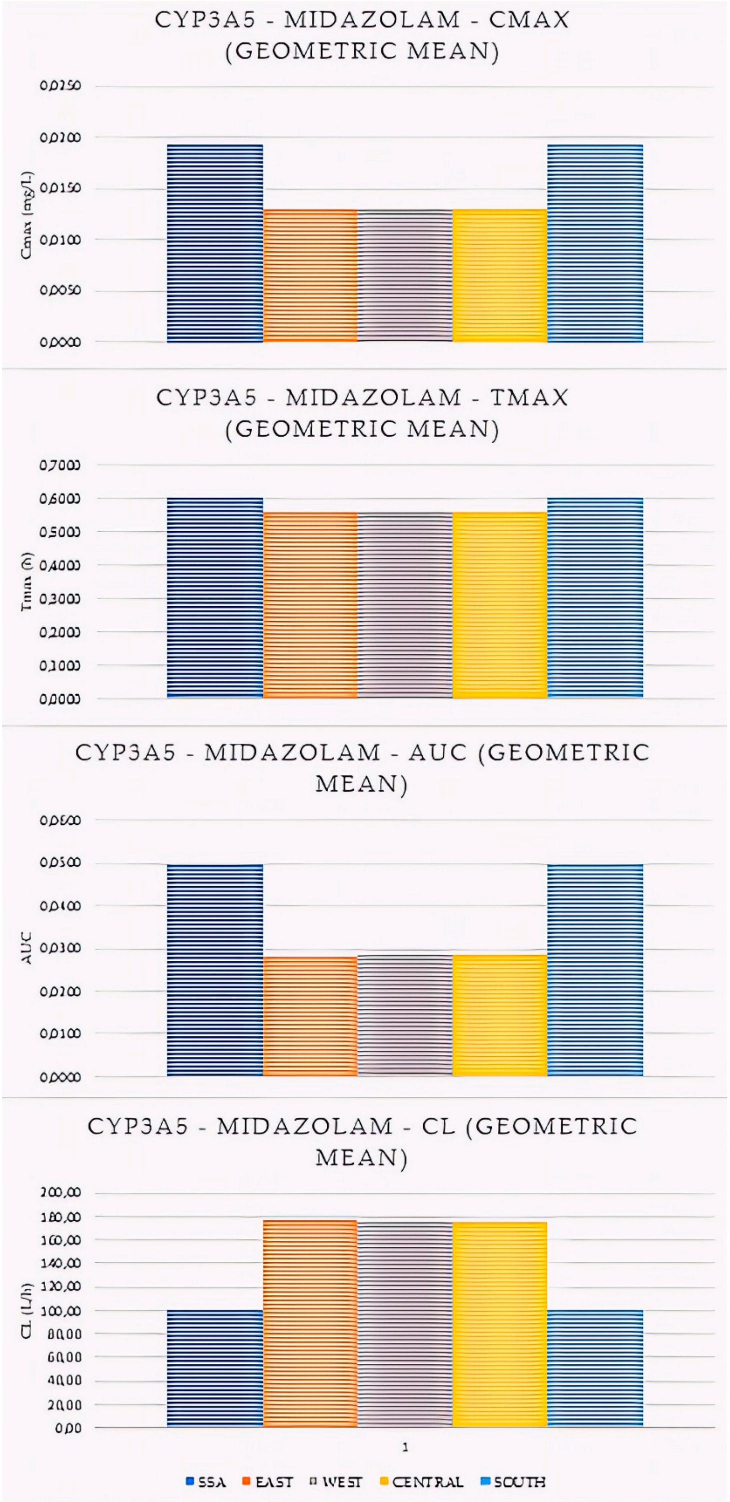

Figure S5: Representation of the pharmacokinetic (PK) parameters C_{max}, T_{max}, AUC_{ss} and CL/F of midazolam after a standard dose of 5 mg o.d. for 30 days. The PK parameters are shown in bar charts representing the 5 regions being Sub Saharan Africa (SSA), East, West, Central and South Africa.
